# Bibliometric analysis of autophagy in NAFLD from 2004 to 2023

**DOI:** 10.1097/MD.0000000000040835

**Published:** 2024-12-06

**Authors:** Sumei Xu, Yating Zhang, Qi Huang, Yiwen Xie, Xiaojuan Tong, Haoge Liu

**Affiliations:** a The First Affiliated Hospital of Zhejiang Chinese Medical University (Zhejiang Provincial Hospital of Chinese Medicine), Hangzhou, China; b Zhejiang Chinese Medical University, Hangzhou, China.

**Keywords:** autophagy, bibliometrics, CiteSpace, NAFLD, VOSviewers

## Abstract

**Background:**

Autophagy is a cellular process in which damaged organelles or unnecessary proteins are encapsulated into double-membrane structures and transported to lysosomes for degradation. Autophagy plays a crucial role in various liver diseases, including nonalcoholic fatty liver disease. This study aims to elucidate the role of autophagy in nonalcoholic fatty liver disease through bibliometric analysis.

**Methods:**

Literature was retrieved from Web of Science CoreCollection database, and the search time was from January 01, 2004 to December 31, 2023. Data retrieval was performed using the Bibliometrix package in R software. VOSviewer and CiteSpace were utilized to visualize the research hotspots and trends related to the effect of autophagy on nonalcoholic fatty liver disease.

**Results:**

A total of 966 papers were obtained, published in 343 journals from 1385 institutions across 57 countries. The journals with the most publications were the “International Journal of Molecular Sciences” and “Scientific Reports.” China had the highest number of published papers. The most productive authors were Yen Paul M and Jung Tae Woo, while Singh R was the most frequently co-cited author. Emerging research hotspots were associated with keywords such as insulin resistance, ferroptosis, endoplasmic reticulum stress, and mitochondrial function.

**Conclusion:**

Research on autophagy in nonalcoholic fatty liver disease is still in its early stages, with a growing body of literature. This study is the first to provide a comprehensive bibliometric analysis, synthesizing research trends and advancements. It identifies current development trends, global cooperation models, foundational knowledge, research hotspots, and emerging frontiers in the field.

## 1. Introduction

nonalcoholic fatty liver disease (NAFLD) is a metabolic disorder of the liver, as well as a progressive liver disease, which usually progresses to liver fibrosis, liver cirrhosis, and hepatocellular carcinoma.^[1]^ Currently, NAFLD is considered a complex metabolic disease caused by the interactions between genetic susceptibility, metabolic disorders, and environmental factors. Associated with type 2 diabetes mellitus (T2DM), dyslipidemia, and cardiovascular disease,^[2]^ NAFLD is a hepatological manifestation of obesity-related metabolic syndrome. In recent years, NAFLD has developed into a worldwide public health issue that poses a threat to human health. Therefore, Epidemic mitigation strategies of NAFLD are urgently needed for global health.

As a strictly regulated process, autophagy is the irreversible degradation of damaged and unwanted components in eukaryotic cells.^[3]^ The initiation and termination of autophagy involves multiple phosphorylation of signaling pathway components, including the BMP/GDF signaling pathway, the mTOR signaling pathway, and the Beclin1-PI3K signaling pathway.^[4–6]^ Three types of autophagy have been identified in mammals: macroautophagy, microautophagy, and chaperone-mediated autophagy. Macroautophagy (hereafter referred to as autophagy) is the most common.^[7]^ Many studies have suggested that NAFLD pathogenesis is mediated by dysregulation of autophagy over the last decade.^[8]^ Thus, regulation of autophagy may serve as a therapeutic target in the treatment of NAFLD.

In recent years, bibliometrics has gained increasing recognition as a method for developing research guidelines and exploring trends in research. An overview of the research field is provided by the bibliometric study using publication indicators such as journals, authors, institutions, and countries.^[9]^ To analyze the scientific knowledge network and evolution in a specific field, bibliometric tools (such as CiteSpace, VOSviewer, Bibliometrix R, Pajek, and Gephi) are used. We will be able to interpret available data more effectively with these new computational tools. However, the field of autophagy for NAFLD lacks any bibliometric studies on cooperation, development trends, or research fronts.

## 2. Materials and methods

### 2.1. Data source and literature search

The literature data was obtained from the Web of Science CoreCollection (WoSCC) database, and the search time was from January 1, 2004 to December 31, 2023. The search formula used was TS= (“nonalcoholic fatty liver” OR “nonalcoholic steatohepatitis” OR “metabolism-related fatty liver” OR NAFLD OR NASH OR MAFLD) AND TS= (“autophagy” OR “macroautophagy” OR “microautophagy” OR “autophagosome” OR “lysosome” OR “autophagic flux” OR “mitophagy” OR “lipophagy” OR “LC3” OR “p62”)

### 2.2. Data screening

#### 2.2.1. Inclusion criteria

Literature related to autophagy and NAFLD; Literature published in English; Literature types include articles and reviews, etc.; and Literature with complete bibliographic information (including title, country, author, keywords, source).

#### 2.2.2. Exclusion criteria

Conference papers, newspapers, patents, achievements, health and popular science literature, etc.; and Duplicate publications.

#### 2.2.3. Data conversion

After screening, the literature was exported in Ref works and plain text formats. Special symbols were removed. Then, the Data Import/Export function in CiteSpace software was used to convert the format of the retrieved literature. Approval from an institutional review board was not required given the bibliometric nature of this study.

#### 2.2.4. Data analysis

VOSviewer (version 1.6.18) was employed as the primary tool for bibliometric analysis in this study. VOSviewer is a versatile software program designed for visualizing and analyzing bibliometric data.^[10]^ It utilizes co-citation and co-occurrence analyses to map relationships among scholarly articles and keywords within a given literature dataset. Co-citation analysis in VOSviewer identifies articles frequently cited together, indicating influential works and key contributors within a research field. This method helps in identifying intellectual networks and seminal contributions. Co-occurrence analysis, on the other hand, identifies clusters of related keywords, revealing prominent research themes and emerging trends. In this study, a comprehensive literature search was conducted to collect relevant articles on the chosen topic. The collected data underwent preprocessing to ensure accuracy and consistency. Subsequently, VOSviewer was used to generate visual maps that visually represent the bibliometric relationships. In the graphical representation, nodes represent terms (e.g., key concepts or author names), and links between nodes indicate co-occurrence relationships based on shared occurrences in the literature. Node size reflects the frequency of occurrence of each term, while link thickness signifies the strength of co-occurrence.^[11]^

CiteSpace is a specialized tool widely used in bibliometric analysis to visualize and analyze citation networks within scholarly literature. CiteSpace integrates temporal and network analyses to identify influential papers, emerging research trends, and significant intellectual structures over time.^[12]^ Unlike traditional citation analysis tools, CiteSpace emphasizes the temporal evolution of research themes, providing insights into the growth and impact of research domains. It enables researchers to uncover pivotal papers and research fronts, facilitating a deeper understanding of scholarly impacts and knowledge dissemination.^[13]^

R, a powerful statistical programming language widely used in scientific research, is instrumental in bibliometric analysis for its robust capabilities in data manipulation, statistical modeling, and visualization.^[14]^ Researchers leverage R’s extensive libraries and packages to preprocess large datasets of scholarly literature, conduct sophisticated statistical analyses, and generate insightful visualizations such as network graphs and plots.

These 3 software tools were utilized to analyze the included literature for publication volume, journals, core author cooperation, and keyword analysis. The time slice period was set to January 2004 to December 2023 (from the first publication of literature within 20 years until now), and the year per slice was set to 1 year.

## 3. Results

### 3.1. Literature search and selection results

**Figure 1.**
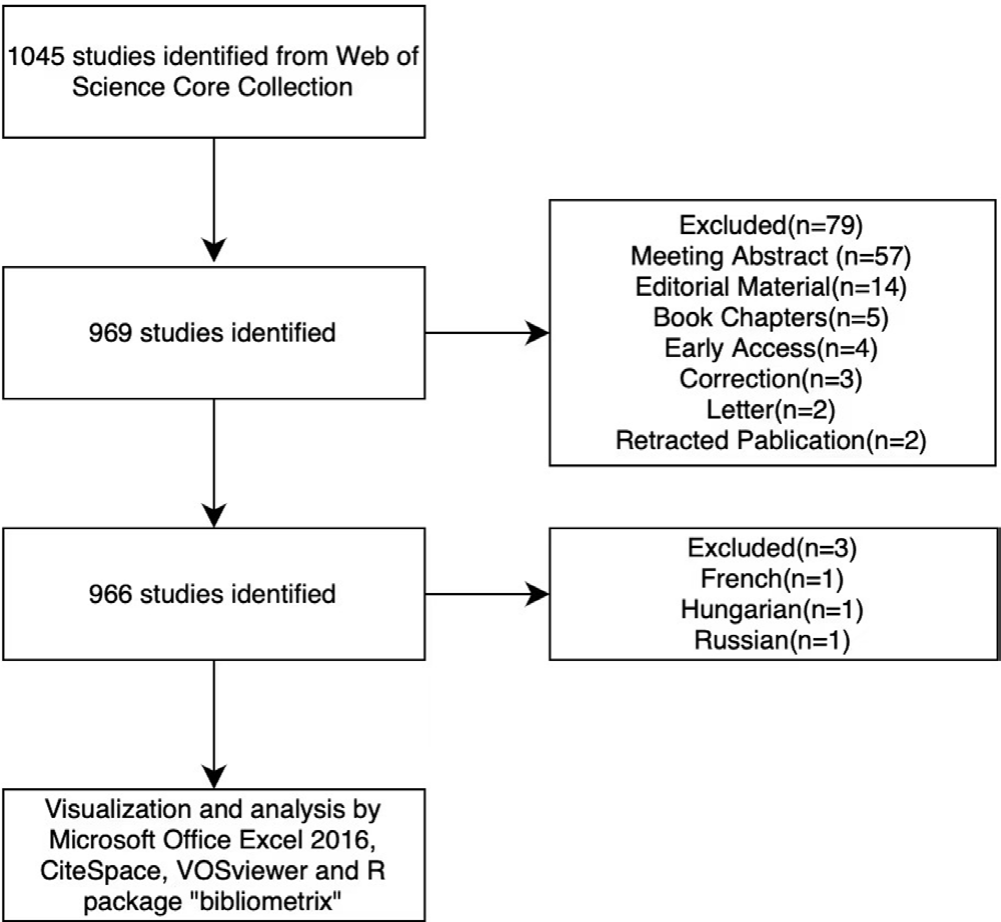
The flowchart of literature data screening.

Through literature search, a total of 1045 articles were retrieved. After removing duplicates and screening for eligibility, 969 articles were selected based on inclusion criteria. The specific screening process is illustrated in Figure 1.

### 3.2. Quantitative analysis of publication

**Figure 2.**
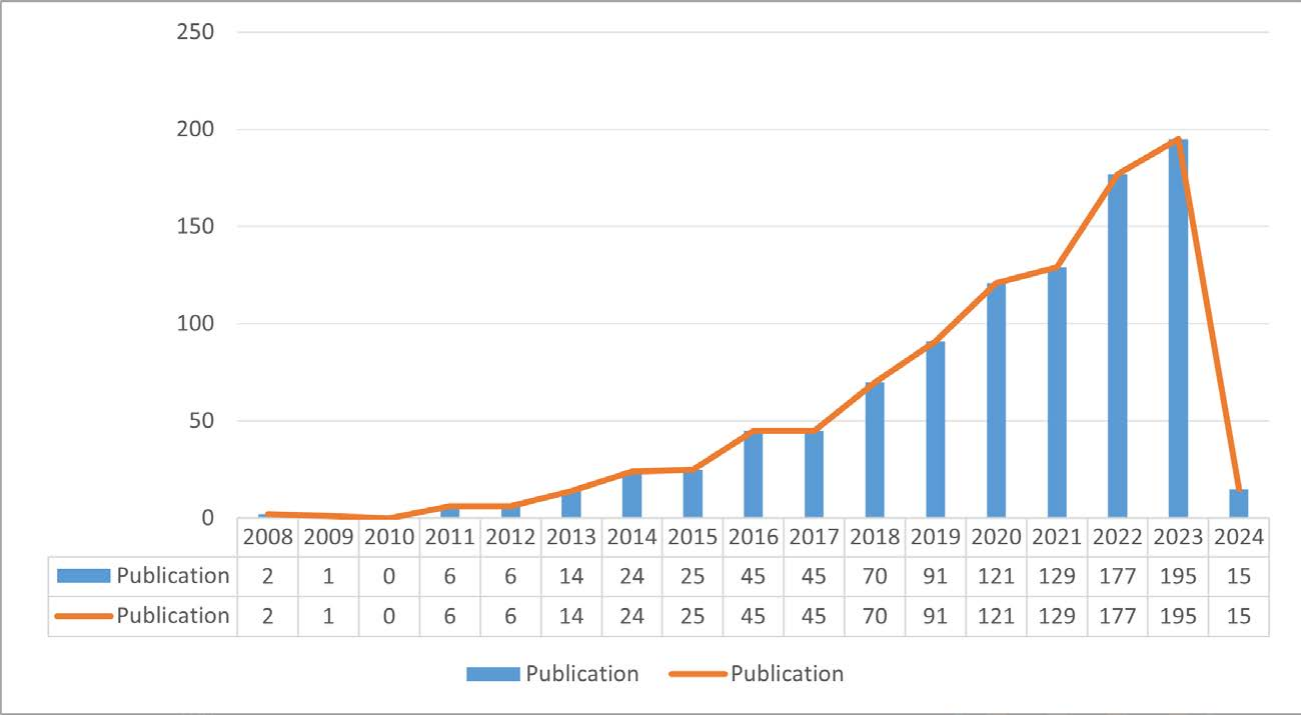
Annual output of research of autophagy in NAFLD. NAFLD = nonalcoholic fatty liver disease.

In total, 966 papers were retrieved through the literature search based on the inclusion criteria, which examined the role of autophagy in the NAFLD. Among these publications, there were 743 research articles and 223 review articles. As illustrated in Figure 2, the frequency of these publications has exhibited a consistent upward trajectory over the past 2 decades. This continuous increase in research output reflects a growing interest in the involvement of cellular autophagy mechanisms in NAFLD. The period spanning from 2004 to 2007 witnessed minimal research on cellular autophagy in NAFLD. From 2008 to 2013, there was a relatively modest publication count, averaging 4.8 publications per year, indicating the early phase of research into cellular autophagy in NAFLD. The limited number of publications during this period suggests that the pertinent theories in this field had not yet been comprehensively validated. The years from 2013 to 2018 experienced a gradual increase in publication volume, marking the developmental phase of research into cellular autophagy in NAFLD. As time progressed, there was a discernible shift in focus within NAFLD research towards cellular autophagy, signifying an increasing Acknowledgments of its significant role. From 2019 onwards, there has been an explosive surge in publications, representing the peak phase of research into cellular autophagy in NAFLD. Notably, the publication count soared in 2022 with 177 papers and in 2023 with 195 papers. This pronounced increase in publications during these 2 years highlights the burgeoning academic interest and research emphasis on cellular autophagy in NAFLD. In summary, the escalating number of publications indicate that the research on cellular autophagy in NAFLD has emerged as a prominent and rapidly evolving field of study.

### 3.3. Country and institutional analysis

**Figure 3.**
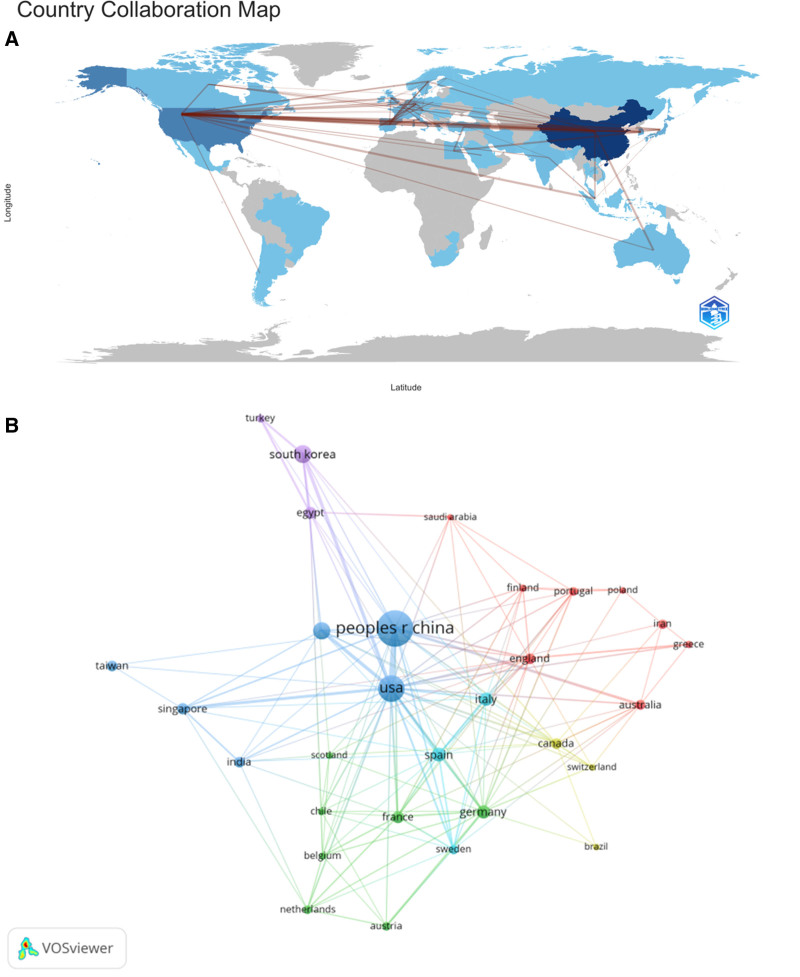
The geographical distribution (A) and visualization of countries (B) on the research of autophagy in NAFLD. NAFLD = nonalcoholic fatty liver disease.

**Figure 4.**
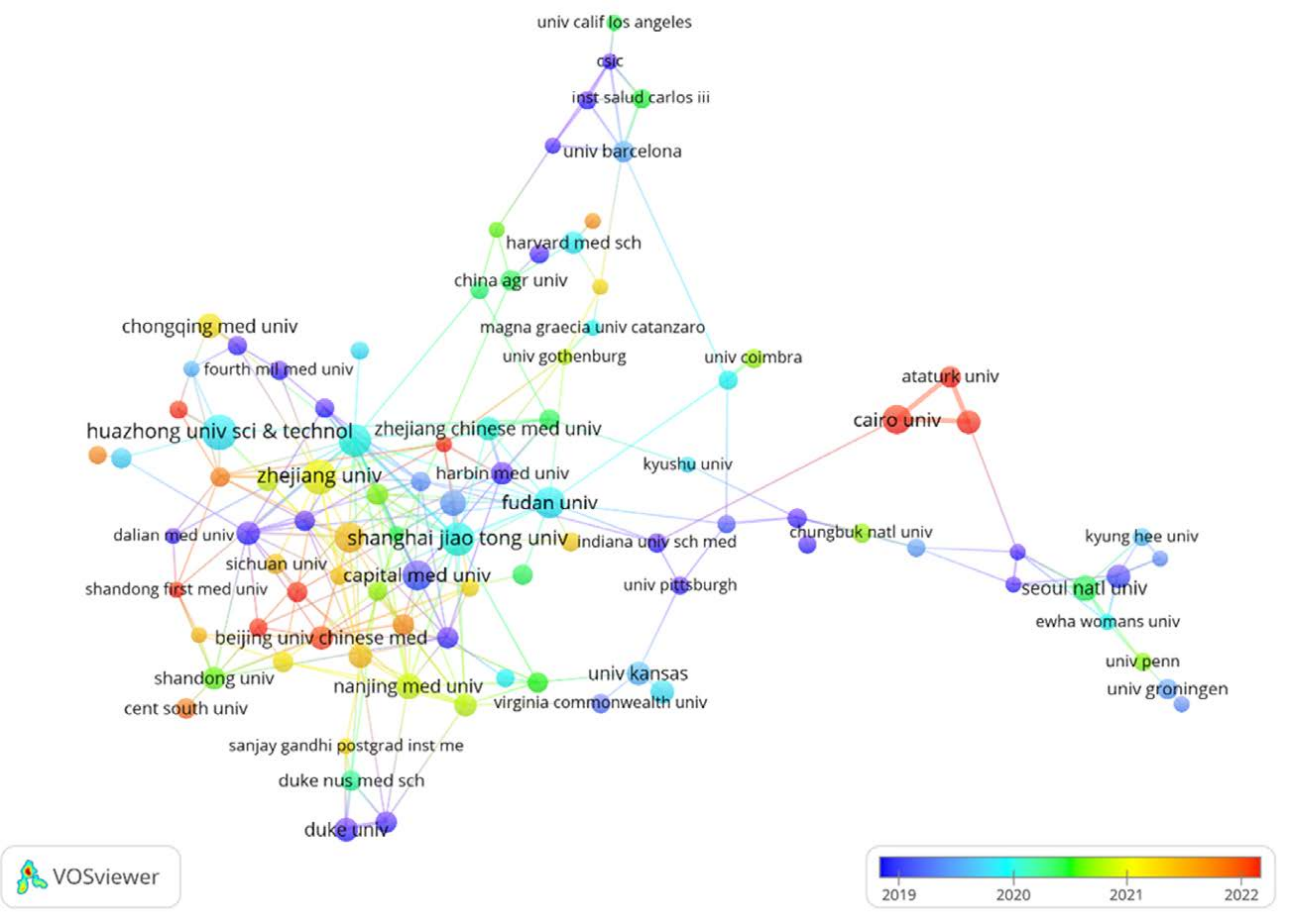
The visualization of institutions on the research of autophagy in NAFLD. NAFLD = nonalcoholic fatty liver disease.

As shown in Table 1, Figure 3. these publications originate from 57 countries and 1385 institutions, with the top 10 countries predominantly situated in Asia, North America, and Europe. Among these nations, China (n = 449, 46.5%) leads in publication count, followed by the United States (n = 124, 12.8%), South Korea (n = 69, 7.1%), and Japan (n = 52, 5.4%). Publications from China and the United States collectively contribute to over half of the total output (58.6%). Subsequently, a collaboration network was constructed based on the publication count and relationships of each country. Notably, positive collaborations exist among different nations, with close cooperation observed between China and the United States. The top 3 institutions publishing relevant papers are: Egypt Knowledge Bank (n = 51), Paris-Cite University (n = 44), and Zhejiang University (n = 38) (Fig. 4).

**Table 1:** Top 10 countries and institutions on the research of autophagy in NAFLD

Rank	Country	Count	Institution	Count
1	CHINA	449	EGYPTIAN KNOWLEDGE BANK	51
2	USA	124	UNIVERSITE PARIS CITE	44
3	KOREA	69	ZHEJIANG UNIVERSITY	38
4	JAPAN	52	CIBER CENTRO DE INVESTIGACION BIOMEDICA EN RED	34
5	ITALY	31	INSTITUT NATIONAL DE LA SANTE ET DE LA RECHERCHE MEDICALE	30
6	SPAIN	29	UNIVERSITY OF MISSOURI COLUMBIA	29
7	GERMANY	25	UNIVERSITY OF BARCELONA	27
8	EGYPT	18	UNIVERSITY OF TSUKUBA	27
9	FRANCE	16	CHINESE ACADEMY OF SCIENCES	26
10	SINGAPORE	16	UNIVERSITY OF MISSOURI SYSTEM	26

NAFLD = nonalcoholic fatty liver disease.

### 3.4. Journal and co-cited journals

**Figure 5.**
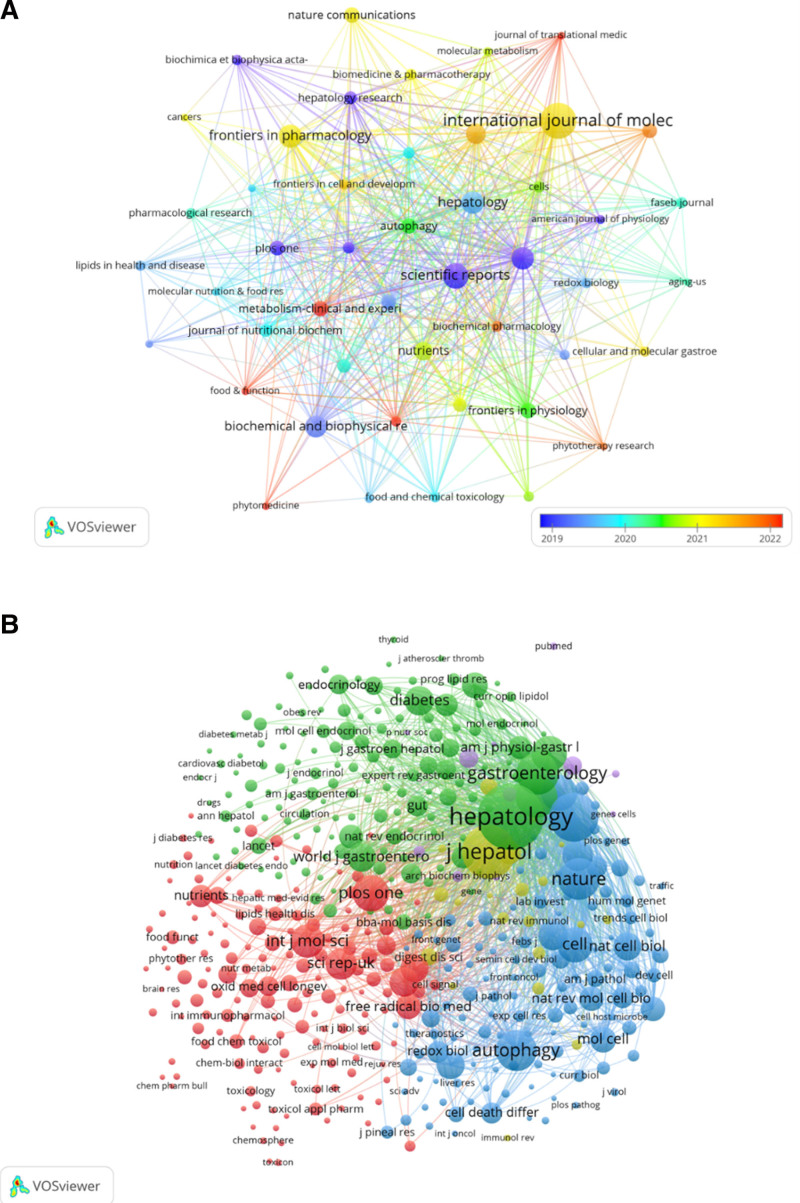
The visualization of journals (A) and co-cited journals (B) on the research of autophagy in NAFLD. NAFLD = nonalcoholic fatty liver disease.

Publications related to cellular autophagy in NAFLD are distributed across 343 journals (Fig. 5). The journal with the highest number of publications is International Journal of Molecular Sciences (n = 46, 4.8%), followed by Scientific Reports (n = 28, 2.9%), and Frontiers in Pharmacology (n = 24, 2.5%). Among the top 15 journals, the one with the highest impact factor is Journal of Hepatology (IF = 26.8), followed by Autophagy (IF = 14.6) (Table 2). Subsequently, we filtered out 47 journals based on the minimum publication count (equal to 5) and constructed a journal network. Among these, Journal of Hepatology, International Journal of Molecular Sciences, Hepatology, Autophagy, and Cell Death & Disease exhibit significant citation relationships. Among the top 15 co-cited journals, journals have been cited over 1000 times, with Hepatology (co-citation = 3418) being the most cited journal, followed by Journal of Biological Chemistry (co-citation = 1875) and Journal of Hepatology (co-citation = 2220). Additionally, Nature has the highest impact factor (IF = 50.5), followed by Cell Metabolism (IF = 27.7). Filtering out journals with a minimum co-citation of 50, we mapped the co-citation network. Journal of Biological Chemistry, Hepatology and Gastroenterology have positive co-citation relationships.

**Table 2:** Top 10 journals and co-cited journals for research of autophagy in NAFLD

Rank	Cited References	Articles	IF	Q	Co-cited journal	Cociattion	IF	Q
1	International Journal of Molecular Sciences	46	4.9	2	Hepatology	3418	12.9	1
2	Scientific Reports	28	3.8	2	Journal of Hepatology	2220	26.8	1
3	Frontiers in Pharmacology	24	4.4	2	Journal of Biological Chemistry	1875	4	2
4	Hepatology	23	12.9	1	Gastroenterology	1344	25.7	1
5	Journal of Hepatology	23	26.8	1	Cell Metabolism	1333	27.7	1
6	Biochemical and Biophysical Research Communications	22	2.5		Nature	1331	50.5	1
7	Life Sciences	19	5.2	2	Autophagy	1288	14.6	1
8	Nutrients	17	4.8	2	P NATL ACAD SCI USA	1003	0.5	4
9	Autophagy	13	14.6	1	PLoS One	973	2.9	3
10	Frontiers in Physiology	13	3.2	3	Journal of Clinical Investigation	957	13.3	1

IF = Impact Factor (2020–2021), NAFLD = nonalcoholic fatty liver disease, P NATL ACAD SCI USA = PROCEEDINGS OF THE NATIONAL ACADEMY OF SCIENCES OF THE UNITED STATES OF AMERICA.

**Figure 6.**
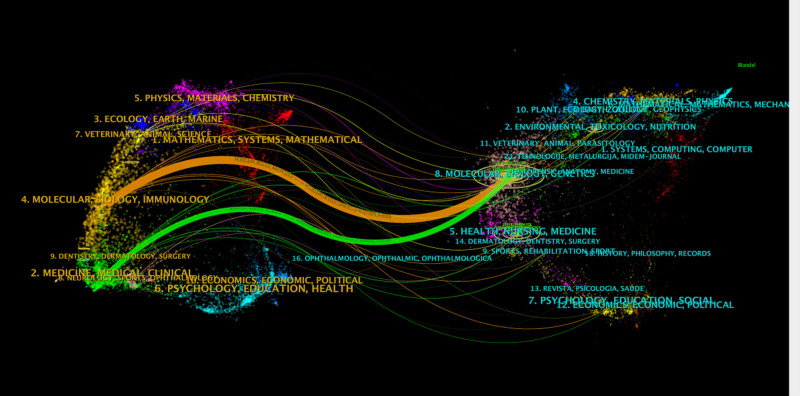
The dual-map overlay of journals on the research of autophagy in NAFLD. NAFLD = nonalcoholic fatty liver disease.

The dual-map overlay of journals displays the citation relationships and academic exchange network between journals and co-cited journals. On the left are co-cited journal clusters, and on the right are citing journal clusters. As shown in Figure 6, the orange paths represent the primary citation pathways, indicating that papers published in molecular/biology/genetics journals are primarily cited by literature in molecular/biology/immunology journals. Through co-cited journal clustering, a deeper analysis of citation relationships among literature can be conducted, thereby gaining insights into the dissemination pathways of important literature and academic influence within the academic field.^[15]^ Co-cited journal clustering illustrates the academic exchange network among different journals. By observing the connections between clusters and the distribution of nodes within clusters, the structure of academic communities and patterns of academic collaboration can be understood.^[16]^

### 3.5. Authors and co-cited authors

**Figure 7.**
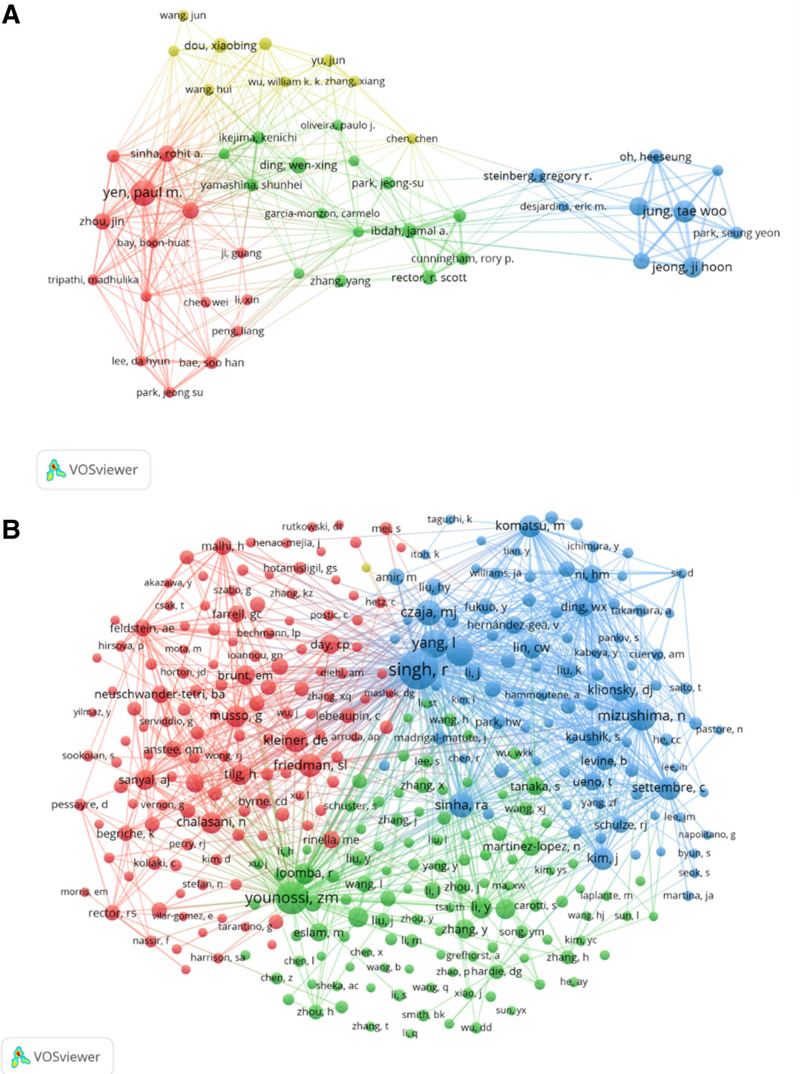
The visualization of authors (A) and co-cited authors (B) on the research of autophagy in NAFLD. The node and line color represented the cluster it belonged to. NAFLD = nonalcoholic fatty liver disease.

A total of 6231 authors have contributed to research on cellular autophagy in nonalcoholic fatty liver disease. Among the top 10 authors, 4 authors have published over 15 papers each (refer to Table 3). We constructed a collaboration network based on authors who have published 5 or more papers (Fig. 7). Yen Paul M, Jung Tae Woo, and Jeong Ji Hoon have the largest nodes due to their significant contributions to related publications. Additionally, we observed concentrated cooperation among authors forming clusters. For instance, Yen Paul M. collaborates actively with others. Among 28,242 co-cited authors, 3 authors have been co-cited over 200 times. The most co-cited author is Singh R (n = 405), followed by Younossi Z (n = 274), and Yang L (n = 203). After filtering authors with a minimum co-citation of 20, we mapped the co-citation network (Fig. 7). Positive collaborations exist among different co-cited authors, such as Singh R, Younossi Z, and others.

**Table 3:** Top 10 authors and co-cited authors on the research of mitochondria in NAFLD

Rank	Authors	Count	Co-cited authors	Citations
1	YEN PM	17	SINGH, R	405
2	JUNG TW	17	YOUNOSSI Z	274
3	JEONG JH	16	YANG L	203
4	ZHANG J	15	CORAZZARI M	176
5	WANG H	14	FIMIA GM	176
6	LI J	13	LO IACONO O	176
7	SINHA RA	13	PIACENTINI M	176
8	ZHANG H	13	VARGAS-CASTRILLÓN J	176
9	ZHANG L	13	AGRA N	174
10	SINGH BK	12	BOSCÁ L	174

NAFLD = nonalcoholic fatty liver disease.

**Figure 8.**
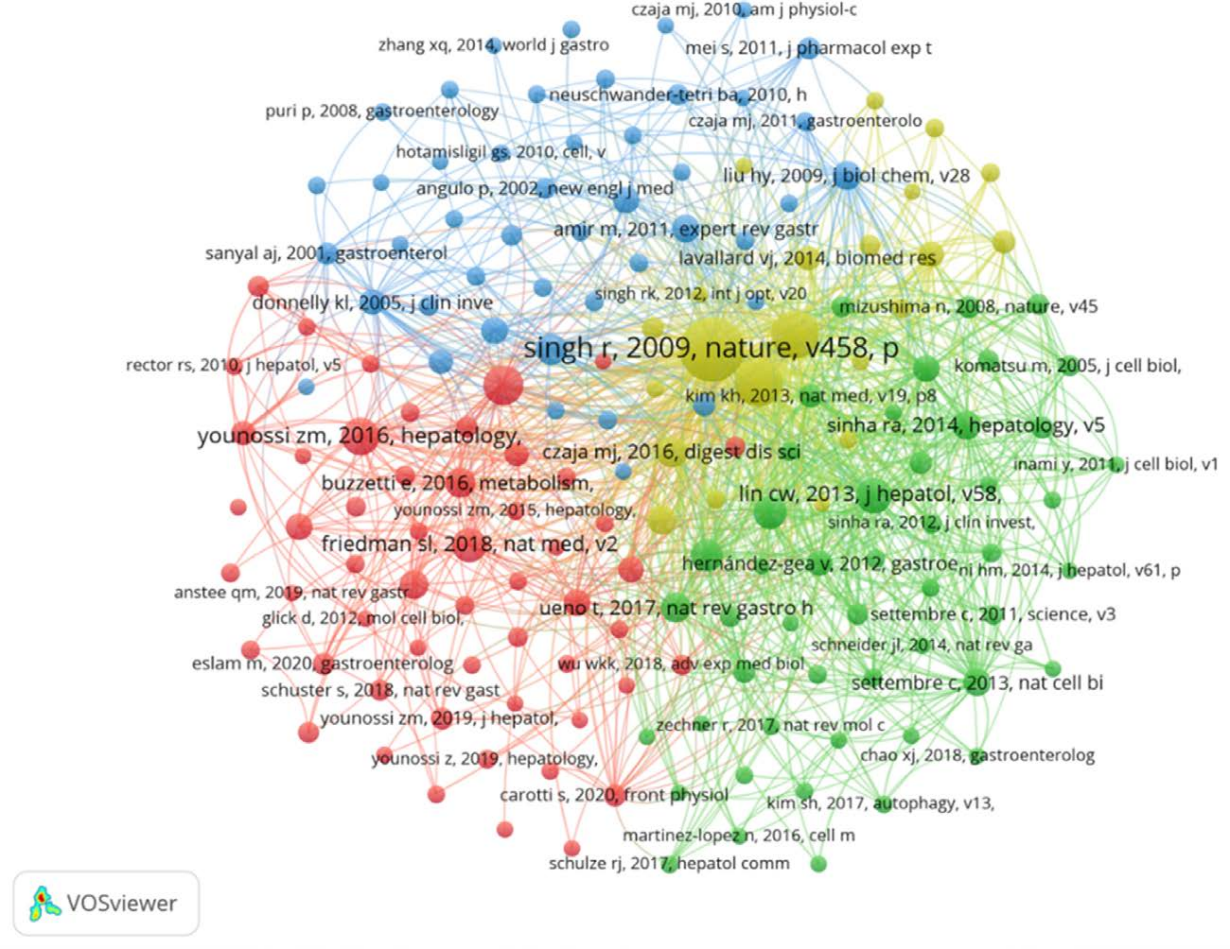
The visualization of co-cited references on the research of autophagy in NAFLD. The node and line color represented the cluster it belonged to. NAFLD = nonalcoholic fatty liver disease.

Over the past 2 decades, there have been 42,113 co-cited references related to nonalcoholic fatty liver disease (NAFLD). Among the top 10 co-cited references (Table 4), all references have been co-cited at least 70 times, with one reference being co-cited a maximum of 328 times. We selected references co-cited 20 times or more to construct a co-citation network (Fig. 8). “Singh R, 2009, Nature, v458, p” demonstrates active co-citation relationships with “Friedman SL, 2018, Nat Med, v2,” “Younossi Z, 2016, Hepatology,” and “Yang L, 2010, Cell Metab, v11.”

**Table 4:** Top 10 co-cited references on the research of autophagy in NAFLD

Rank	Cited references	Citations
1	SINGH R, 2009, NATURE, V458, P1131, DOI 10.1038/NATURE07976	328
2	YANG L, 2010, CELL METAB, V11, P467, DOI 10.1016/J.CMET.2010.04.005	176
3	KLEINER DE, 2005, HEPATOLOGY, V41, P1313, DOI 10.1002/HEP.20701	174
4	YOUNOSSI ZM, 2016, HEPATOLOGY, V64, P73, DOI 10.1002/HEP.28431	123
5	LIN CW, 2013, J HEPATOL, V58, P993, DOI 10.1016/J.JHEP.2013.01.011	111
6	FRIEDMAN SL, 2018, NAT MED, V24, P908, DOI 10.1038/S41591-018-0104-9	94
7	KIM J, 2011, NAT CELL BIOL, V13, P132, DOI 10.1038/NCB2152	92
8	TANAKA S, 2016, HEPATOLOGY, V64, P1994, DOI 10.1002/HEP.28820	82
9	CZAJA MJ, 2016, DIGEST DIS SCI, V61, P1304, DOI 10.1007/S10620-015-4025-X	77
10	DAY CP, 1998, GASTROENTEROLOGY, V114, P842, DOI 10.1016/S0016-5085(98)70599-2	71

NAFLD = nonalcoholic fatty liver disease.

### 3.6. Burst citation references analysis

**Figure 9.**
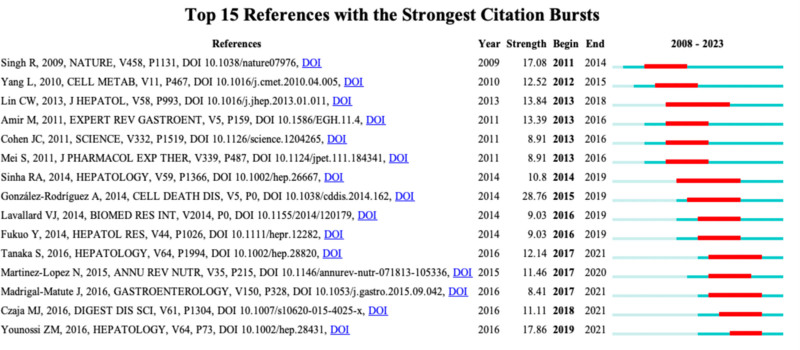
Top 15 references with strong citation bursts. A red bar indicates high citations in that year.

Burst citation references refer to those references that are frequently cited by scholars in a specific field within a certain period. In our study, CiteSpace identified 15 references with strong burst citation. Each bar represents a year, with red bars indicating intense burst citations (Fig. 9). Burst citations for references occurred from 2011 to 2021. The reference with the strongest burst citation (intensity = 28.76) is titled “Impaired autophagic flux is associated with increased endoplasmic reticulum stress during the development of NAFLD,” authored by Á González-Rodríguez et al., with burst citations spanning from 2015 to 2019. The second strongest burst citation (intensity = 17.86) is for the reference titled “Autophagy regulates lipid metabolism,” authored by Younossi et al, with burst citations from 2019 to 2021. Overall, the burst intensity of these 15 references ranges from 8.91 to 28.76, with a duration of persistence ranging from 3 to 6 years. Table 5 summarizes the main research contents of the top 15 burst citation references in order.

**Table 5:** The main research contents of the 15 references with strong citations bursts

Rank	Strength	Main research content
1	17.08	Autophagy regulates lipid storage by interacting with lipid droplets during nutrient deprivation, suggesting a new therapeutic avenue for metabolic diseases associated with lipid accumulation.
2	12.52	Autophagy is an important regulator of organelle function and insulin signaling, and the loss of autophagy is a critical component of the defective insulin action seen in obesity
3	13.84	Enhancing macroautophagy pharmacologically reduces fatty liver and liver damage, whereas inhibiting it exacerbates these conditions.
4	13.39	Autophagy influences the progression of nonalcoholic steatohepatitis (NASH), suggesting therapeutic potential in enhancing this process.
5	8.91	Exploring the mechanisms of NAFLD from the perspectives of human genetic and metabolic studies.
6	8.91	The data show that forming triglyceride-rich lipid droplets and inducing autophagy protect against lipotoxicity, as evidenced by autophagy-associated hepatic steatosis in high-fat diet-fed mice.
7	10.8	Caffeine reduces the risk of nonalcoholic fatty liver disease (NAFLD) by enhancing hepatic autophagy and fat oxidation, effectively decreasing liver fat in high-fat diet-fed mice.
8	28.76	autophagic flux is impaired in the liver from both NAFLD patients and murine models of NAFLD, as well as in lipid-overloaded human hepatocytes, and it could be due to elevated ER stress leading to apoptosis
9	9.03	Current understanding of autophagy and its role in the progression of hepatic complications associated with obesity, ranging from steatosis to hepatocellular carcinoma.
10	9.03	The decrease in hepatic cathepsin expression in NAFLD is associated with autophagic dysfunction. Hepatic inflammation correlates with autophagic dysfunction in NAFLD
11	12.14	Rubicon is overexpressed and plays a pathogenic role in NAFLD by accelerating hepatocellular lipoapoptosis and lipid accumulation, as well as inhibiting autophagy
12	11.46	This review examines lipophagy’s molecular regulation, including autophagosome targeting of lipid droplets, ATG protein involvement in membrane curvature, interactions with cytosolic lipases, and implications for fatty liver disease.
13	8.41	It discusses some of these connections and how hepatic autophagy might serve as a therapeutic target in common metabolic disorders.
14	11.11	The roles of autophagy in liver function, coupled with reduced hepatic autophagy in conditions like obesity and aging linked to NAFLD, indicate autophagy as a potential therapeutic target for this disease.
15	17.86	Meta-analysis assessing the global epidemiology of nonalcoholic fatty liver disease, including prevalence, incidence, and outcomes.

### 3.7. Hotspots and frontiers

**Figure 10.**
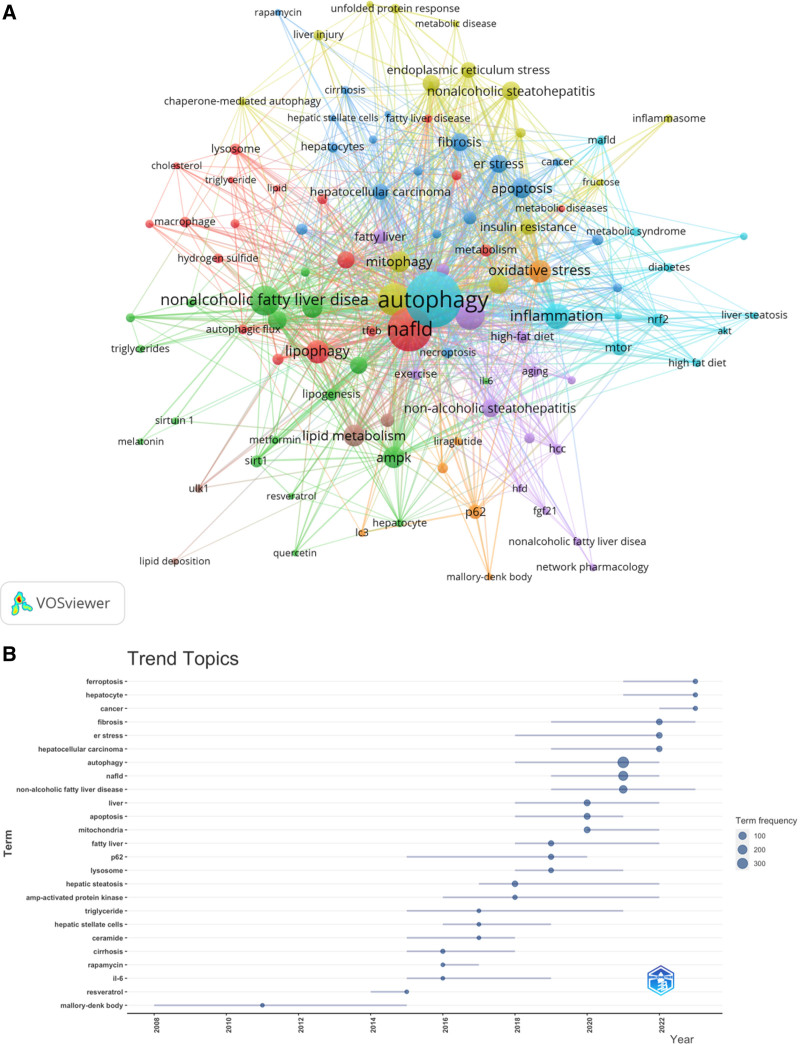
Keyword cluster analysis (A) and trend topic analysis (B) In A, the node and line color represented the cluster it belonged to.

Keyword co-occurrence analysis enables the rapid identification of research hotspots in a particular field. Table 6 presents the top 10 high-frequency keywords in the study of cellular autophagy in nonalcoholic fatty liver disease. Among these keywords, terms such as insulin resistance, oxidative stress, and endoplasmic reticulum stress are included, representing future research directions of autophagy in NAFLD. We filtered keywords appearing 5 times or more and conducted cluster analysis using VOSviewer (Fig. 10). The thickness of the lines between nodes indicates the strength of connections between keywords. We obtained a total of 8 clusters, representing 5 research directions. Keywords in the green cluster include melatonin, metformin, quercetin, lipid metabolism, etc. Keywords in the red cluster include cholesterol, triglycerides, autophagy, hydrogen sulfide, fatty liver, etc. Keywords in the blue cluster include cellular autophagy, inflammation, hepatocellular carcinoma, metabolic syndrome, high-fat diet, hepatic steatosis, NRF2, mTOR, etc. Keywords in the purple cluster include aging, network pharmacology, etc. Keywords in the yellow cluster include endoplasmic reticulum stress, protein response, liver injury, cell engulfment, oxidative stress, etc.

**Table 6:** Top 10 high‐frequency keywords

Rank	Keywords	Counts
1	autophagy	294
2	fatty liver-disease	201
3	insulin-resistance	190
4	oxidative stress	156
5	activation	154
6	expression	127
7	obesity	119
8	metabolism	108
9	endoplasmic-reticulum stress	101
10	steatosis	99

Keyword trend analysis indicates that from 2014 to 2020, research mainly focused on metabolism and gene expression, with primary keywords including apoptosis, mitochondria, p62, hepatic steatosis, protein kinase, triglycerides, and ceramide. Since 2021, scholars have actively explored the pathogenesis and therapeutic potential of cellular autophagy in nonalcoholic fatty liver disease, with cellular autophagy treatment becoming mainstream. Key topics include autophagy, nonalcoholic fatty liver disease, hepatocellular carcinoma, stress, fibrosis, and ferroptosis. Furthermore, the keywords non-fibrosis, stress, and ferroptosis have been frequently appearing in the past 4 years (2020–2023), suggesting they likely represent current research hotspots in cellular autophagy in NAFLD.

## 4. Discussion

In this study, we employed CiteSpace, VOSviewer software, and R language techniques to perform a visual analysis of 966 papers on autophagy in the field of nonalcoholic fatty liver disease from the Web of Science database, spanning the years 2004 to 2024. By generating knowledge maps, we aim to provide researchers with a comprehensive understanding of the publication trends, journal sources, key researchers, institutional collaboration networks, current state, research hotspots, and development trends in this field over the past 20 years.

### 4.1. Current research status

The results of this study indicate that the overall number of publications in this research field is on the rise. The primary publishing countries are China, the United States, South Korea, and Japan. The journals with the highest number of publications are the International Journal of Molecular Sciences and Scientific Reports. The most prolific authors are Yen Paul M and Jung Tae Woo. The top 3 research institutions in terms of the number of published papers are the Egyptian Knowledge Bank, Paris-Saclay University, and Zhejiang University. Most of these research institutions are universities from various countries, showing some level of collaboration among them. However, it is noteworthy that Chinese institutions often limit their collaborations to domestic partners rather than engaging in international exchanges. This inward-focused approach may be detrimental to the long-term development of academic research.

In terms of annual publication volume, the total number of publications in this field is increasing, with China, the United States, and France being the main publishing countries. Our findings suggest that research outcomes related to autophagy in NAFLD have seen rapid development in recent years,^[8,17]^ showing significant progress compared to other fields. Nevertheless, there is a notable lack of international and intercontinental collaboration among institutions, and the sharing of research outcomes is insufficient. Moving forward, it is essential to enhance collaboration and share research results across institutions to foster in-depth development in this field.

### 4.2. Hotspots and frontiers

References with citation bursts can reflect the evolution and emerging trends in a scientific field, as these papers are frequently cited by researchers in the field. The current hotspots in autophagy research within the context of NAFLD focus on the expression of autophagy regulatory factors, endoplasmic reticulum stress, lipid metabolism, and changes in the level of steatosis-induced damage.^[18–21]^ These research areas collectively represent the cutting-edge of this field, highlighting the importance of elucidating the complex relationship between autophagy and the pathogenesis of NAFLD, with the ultimate goal of translating these insights into innovative therapeutic approaches for NAFLD.

Keywords are also a crucial method for identifying the distribution and evolution of research hotspots in a field. Through keyword clustering and thematic analysis, we have identified that the main research directions of autophagy in the context of NAFLD focus on the following areas: insulin resistance, ferroptosis, and endoplasmic reticulum stress, and mitochondrial function.

### 4.3. Insulin resistance

Insulin resistance plays a crucial role in hepatic steatosis, particularly in the context of steatohepatitis.^[22–24]^ It has been reported that patients with NAFLD are at a heightened risk of developing type 2 diabetes mellitus.^[25]^ A systematic review and meta-analysis indicated that over a 5-year follow-up period, NAFLD is associated with approximately a two-fold increased risk of developing T2DM and metabolic syndrome.^[26]^ Additionally, another study demonstrated that nearly all patients with T2DM exhibit nonalcoholic steatohepatitis (NASH), regardless of their liver enzyme levels, highlighting steatohepatitis as a prominent feature of liver damage in T2DM patients.^[27]^

Furthermore, TNF-α significantly contributes to the development of systemic insulin resistance.^[28–30]^ Elevated TNF-α mRNA expression in the liver and adipose tissue of patients with NASH suggests that TNF-α upregulation in adipose tissue may exacerbate NAFLD by increasing systemic insulin resistance and promoting inflammation across various tissues.^[31,32]^ Similarly, increased secretion of IL-6 in the visceral adipose tissue of obese individuals has been noted.^[33]^ In vitro studies using primary mouse hepatocytes and human hepatocarcinoma cells have shown that IL-6 induces insulin resistance through the phosphorylation of STAT3, mediated by SOCS-3 and regulated by mTOR.^[34]^ The application of Rapamycin, a specific inhibitor of mTOR, has been found to improve IL-6-induced insulin resistance in hepatocytes.^[35]^ Notably, serum IL-6 levels are significantly elevated in patients with NAFLD and NASH compared to healthy individuals,^[36]^ linking chronic low-grade inflammation mediated by IL-6 in adipose tissue with the progression of NAFLD.^[37]^

Skeletal muscle, a primary target organ for insulin action, plays a crucial role in maintaining glucose homeostasis and is an early and critical site for the development of insulin resistance.^[38,39]^ Studies involving muscle-specific insulin receptor knockout mice have shown that despite maintaining normal blood glucose levels, these mice exhibit increased fat mass, along with elevated serum triglycerides and free fatty acids.^[40]^ In muscle-specific insulin receptor knockout mice, insulin-stimulated glucose transport and glycogen synthesis in muscle are diminished, while insulin-stimulated glucose transport in adipose tissue is enhanced,^[41]^ indicating that selective inactivation of muscle insulin signaling prompts a substrate redistribution from muscle to adipose tissue.

Additionally, Them2 plays a key role by hydrolyzing long-chain fatty acyl coenzyme A esters into free fatty acids and coenzyme A, predominantly expressed in the liver and oxidative tissues.^[42]^ Studies demonstrate insulin resistance and upregulation of Them2 in the skeletal muscle of wild-type mice fed a high-fat diet.^[43]^ Skeletal muscle-specific Them2 knockout mice fed the same diet showed reduced weight gain, improved glucose homeostasis, and enhanced insulin sensitivity.^[44]^ Them2 influences skeletal muscle metabolism, affecting various metabolic products and modulating myocellular cytokines involved in insulin responsiveness, suggesting its potential as a target for NAFLD treatment. A study found that liver steatosis in NAFLD patients was primarily linked to insulin resistance in skeletal muscle rather than in the liver.^[45]^ Moreover, in a prospective observational cohort study, even after adjusting for factors like insulin resistance and inflammation, individuals with low muscle mass had a higher risk of developing NAFLD.^[46]^ In a biopsy-confirmed NAFLD cohort, muscle loss was significantly associated with NASH and substantial fibrosis, independent of obesity, inflammation, and insulin resistance.^[47]^

### 4.4. Ferroptosis

Ferroptosis, a non-apoptotic form of programmed cell death, is characterized by the accumulation of free radicals, oxygen, and iron ions. It also involves the stimulation of the mitogen-activated protein kinase pathway, decreased uptake of cysteine, and depletion of glutathione.^[48,49]^ Recent studies have indicated that approximately one-third of NAFLD patients exhibit elevated liver iron levels, rendering the liver highly susceptible to oxidative damage.^[50]^ This excessive accumulation of iron and resultant oxidative stress are pivotal factors initiating liver injury and disease progression in NAFLD,^[51]^ thereby establishing a close association between ferroptosis and NAFLD.

During ferroptosis, lipid peroxidation inflicts damage on liver cells, which in turn consumes intracellular antioxidants, perpetuating a cycle of oxidative stress.^[52]^ Research including in vivo experiments has demonstrated that treatment with RSL-3, an inducer of ferroptosis, diminishes the expression of glutathione peroxidase 4 in the liver,^[53]^ while increasing 12/15-lipoxygenase and apoptosis-inducing factor levels. These changes underscore the crucial role of ferroptosis in NASH-related lipid peroxidation.^[54]^ Conversely, the iron death inhibitor liproxstatin-1 has been shown to mitigate hepatic lipid peroxidation and related cell death, thus alleviating the severity of NASH.^[55]^ Similarly, quercetin, a natural antioxidant, can inhibit ferroptosis in a high-fat diet-induced model, providing relief from NAFLD.^[56]^

The transcription factor Nrf2, which regulates antioxidant responses, is notably implicated in reducing susceptibility to ferroptosis.^[57]^ High-fat diets decrease Nrf2 expression via the AKT/mTOR/ATG7 pathway, with significant reductions observed in ATG gene knockout mice, leading to enhanced ferroptosis.^[58]^ However, upregulation of Nrf2 expression not only inhibits ferroptosis but also improves outcomes in NAFLD. Ginkgolide B, extracted from Ginkgo biloba leaves, effectively increases Nrf2 expression,^[58,59]^ suggesting a potential treatment avenue for NAFLD by targeting lipid accumulation and oxidative stress-induced ferroptosis through the Nrf2 signaling pathway.^[60]^

Elevated serum ferritin levels are observed in advanced stages of NAFLD, serving as a marker for hepatocellular injury.^[61]^ The higher levels of iron accumulation associated with advanced NAFLD can induce severe lipid peroxidation, which triggers further iron deposition and cell death.^[62]^ In NAFLD models, a high-iron diet exacerbates oxidative stress and inflammation, accelerating the progression of NASH.^[63,64]^ Additionally, the induction of pronin2, a pentapeptide protein associated with lipid homeostasis control, in response to iron deposition suggests a novel pathway to mitigate the effects of iron overload. This protein promotes the synthesis of transferrin, reducing intracellular iron levels and thus rendering cells less susceptible to ferroptosis.^[65]^ This finding points to excessive iron accumulation not only triggering oxidative stress and mitochondrial damage but also impairing pancreatic β-cell function.

### 4.5. Endoplasmic reticulum stress

The endoplasmic reticulum (ER) is one of the largest subcellular compartments, essential for various biological functions including protein folding, calcium homeostasis, and lipid biogenesis.^[66]^ It operates as a network facilitating vesicular transport, and its dysfunction – via the activation of ER stress signaling – plays a significant role in the pathogenesis of metabolically driven nonalcoholic fatty liver disease.

To manage ER stress, eukaryotic cells activate the unfolded protein response (UPR), commonly referred to as the “protein folding response.” During mild to moderate ER stress, the UPR initiates processes to eliminate unfolded or misfolded proteins, thereby restoring ER homeostasis.^[67]^ This adaptive and cytoprotective mechanism is termed the “adaptive/cytoprotective” UPR.^[68]^ However, in cases of severe or prolonged ER stress, the UPR may become hyperactivated, leading to the initiation of intrinsic apoptotic pathways.^[69]^ This response is known as the “maladaptive/unchecked/terminal” UPR.

The UPR is primarily triggered by ER transmembrane proteins such as PERK, IRE1α, and others. Recent research has underscored the pivotal roles of these ER stress and UPR sensors in the development of hepatic steatosis.^[70]^ For instance, activated IRE1α enhances the transcription of serine palmitoyl transferase genes via XBP1s, facilitating the biosynthesis of ceramides and the release of extracellular vehicles from hepatocytes.^[71]^ In diet-induced NASH models, these EVs attract macrophages to the liver, exacerbating inflammation and furthering liver injury associated with diet-induced steatohepatitis.

Recent experimental evaluations have also explored the effects of short-term PERKα treatment on lipid metabolism and liver damage in obese mice.^[72]^ Although no significant changes were noted in the overall lipid profiles of these mice, a reduction in triglyceride content in their livers was observed. However, total cholesterol levels remained unchanged. Mechanistically, PERKα treatment upregulated the expression of genes such as Atf3, Ddit3, and Gdf15, and induced an increase in serum GDF15 levels, potentially contributing to reduced food intake.^[73,74]^ These observations suggest that short-term PERKα treatment may enhance metabolic homeostasis and offer a preventive approach against the development of NAFLD.

### 4.6. Mitochondrial function

Mitochondria are essential organelles that serve as the energy powerhouses of eukaryotic cells. Their primary function is to generate adenosine triphosphate through the process of oxidative phosphorylation, providing the necessary energy for a variety of cellular activities.^[75]^ Beyond energy production, mitochondria are integral to several other cellular processes including the regulation of metabolic pathways, calcium signaling, apoptosis, and the generation of reactive oxygen species (ROS).^[76]^ In liver cells, or hepatocytes, the role of mitochondria is particularly significant due to the liver’s central role in metabolic regulation.^[77]^ Hepatocytes have a high demand for ATP, necessary to fuel various metabolic processes including gluconeogenesis, the synthesis of plasma proteins, and detoxification. Mitochondria in hepatocytes are involved in fatty acid β-oxidation, the primary pathway for the catabolism of fatty acids to produce energy. This process is crucial for maintaining energy homeostasis and for the regulation of lipid levels in the liver.^[78]^ Moreover, mitochondria in liver cells are pivotal in managing ROS levels, which, if not properly controlled, can lead to oxidative stress and cellular damage.^[79]^

In the context of nonalcoholic fatty liver disease, mitochondrial function becomes critically relevant. NAFLD is characterized by excessive fat accumulation in the liver, primarily in the form of triglycerides. This accumulation can lead to mitochondrial dysfunction, which in turn plays a pivotal role in the progression of the disease.^[80]^ Initially, mitochondrial dysfunction may manifest as impaired fatty acid oxidation.^[81]^ This impairment can cause an increase in the intrahepatic triglyceride content, further exacerbating the fat accumulation in the liver. Additionally, dysfunctional mitochondria may produce excessive ROS as a byproduct of impaired electron transport chain activity.^[82]^ This overproduction of ROS can induce oxidative stress, which damages cellular proteins, lipids, and DNA, contributing to hepatocyte injury and inflammation.^[83]^ Moreover, mitochondrial dysfunction in NAFLD can promote the release of pro-inflammatory cytokines that exacerbate liver inflammation and fibrosis.^[84]^ This inflammatory response can further impair mitochondrial function, creating a vicious cycle of worsening liver damage. As NAFLD progresses to its more severe form, NASH, the role of mitochondria becomes even more critical. NASH is associated with significant mitochondrial abnormalities, including altered morphology, reduced number, and impaired function.^[85]^

The link between mitochondrial dysfunction and NAFLD underscores the potential of therapeutic strategies aimed at restoring or supporting mitochondrial function as a means to prevent or mitigate the progression of liver disease. Future research into the mechanisms of mitochondrial dysfunction in NAFLD could provide new insights into the treatment and management of this increasingly common disorder.

### 4.7. Limitation

Firstly, the scope of this study does not include non-English literature, which may introduce bias in the data sources used. Future efforts should broaden the scope of literature searches to include high-quality basic research and multicenter real-world data studies as much as possible. Additionally, experts have recently reached a consensus that metabolic dysfunction associated fatty liver disease (MAFLD) may be a more accurate concept compared to NAFLD.^[86]^ This presents a challenge for researchers, and future bibliometric studies should consider incorporating these new terms to ensure the completeness of search results. Finally, there is still a lack of systematic research in this field, and future efforts should integrate techniques such as bioinformatics and molecular targeted regulation to provide strong theoretical support for the treatment of NAFLD.

## 5. Conclusion

In this study, we conducted a visual analysis of literature related to autophagy and NAFLD using bibliometric tools. The study provided a comprehensive overview of the current status, hot topics, and trends in this field over the past 20 years, identifying key contributors, regions, and publications. Analysis of annual publications revealed an increasing awareness among global scholars regarding the significance of autophagy in NAFLD. While China and the United States are at the forefront in this field, there is still a need for further strengthening of collaboration and exchange among countries and institutions. Visual analysis and research trends indicate that recent hotspots in autophagy and NAFLD research have focused on insulin resistance, ferroptosis, and endoplasmic reticulum stress. This study timely guides future research directions and trends in this field.

## Author contributions

**Conceptualization:** Haoge Liu.

**Data curation:** Sumei Xu, Haoge Liu.

**Formal analysis:** Haoge Liu.

**Funding acquisition:** Xiaojuan Tong.

**Methodology:** Yating Zhang, Yiwen Xie, Xiaojuan Tong.

**Project administration:** Sumei Xu, Yiwen Xie, Xiaojuan Tong.

**Resources:** Yating Zhang.

**Software:** Sumei Xu, Qi Huang, Xiaojuan Tong.

**Supervision:** Yating Zhang, Qi Huang, Yiwen Xie.

**Validation:** Sumei Xu, Yating Zhang, Qi Huang, Yiwen Xie.

**Visualization:** Sumei Xu.

**Writing—original draft:** Haoge Liu.

**Writing—review & editing:** Qi Huang, Yiwen Xie.
